# Research on the Error Characteristics of a 110 kV Optical Voltage Transformer under Three Conditions: In the Laboratory, Off-Line in the Field and During On-Line Operation

**DOI:** 10.3390/s16081303

**Published:** 2016-08-16

**Authors:** Xia Xiao, Haoliang Hu, Yan Xu, Min Lei, Qianzhu Xiong

**Affiliations:** 1 School of Electrical and Electronic Engineering, Huazhong University of Science and Technology, Wuhan 430070, China; xuyan919@hust.edu.cn; 2 State Grid Electric Power Research Institute, Wuhan 430070, China; huhaoliang@epri.sgcc.com.cn (H.H.); leimin@epri.sgcc.com.cn (M.L.); xiongqianzhu@epri.sgcc.com.cn (Q.X.)

**Keywords:** OVT, error characteristics, laboratory, field off-line, on-line operation

## Abstract

Optical voltage transformers (OVTs) have been applied in power systems. When performing accuracy performance tests of OVTs large differences exist between the electromagnetic environment and the temperature variation in the laboratory and on-site. Therefore, OVTs may display different error characteristics under different conditions. In this paper, OVT prototypes with typical structures were selected to be tested for the error characteristics with the same testing equipment and testing method. The basic accuracy, the additional error caused by temperature and the adjacent phase in the laboratory, the accuracy in the field off-line, and the real-time monitoring error during on-line operation were tested. The error characteristics under the three conditions—laboratory, in the field off-line and during on-site operation—were compared and analyzed. The results showed that the effect of the transportation process, electromagnetic environment and the adjacent phase on the accuracy of OVTs could be ignored for level 0.2, but the error characteristics of OVTs are dependent on the environmental temperature and are sensitive to the temperature gradient. The temperature characteristics during on-line operation were significantly superior to those observed in the laboratory.

## 1. Introduction

Optical Voltage Transformers (OVTs) based on the linear electro-optic effect and applied at a certain scale around the world are recognized as a current development trend in the power transformer sector because of their simpler insulation structure, greater security and anti-electromagnetic interference properties [1,2,3]. OVTs developed by Nxtphase (Vancouver, BC, CA) have been applied to voltage levels from 123 kV to 500 kV [4,5], and OVTs developed by NAE (Beijing, China) have been applied to voltage levels from 110 kV to 500 kV [6].

OVTs are one of the electric energy measurement data sources in the power grid, and their measurement performance determines the accuracy and fairness of electric energy metering, therefore, the accuracy calibration and the temperature characteristics of OVTs must be tested before they leave the factory, and the accuracy of OVTs should be calibrated again before being put into operation on-site [7,8].

However, OVTs can be influenced by the ambient temperature. In previous studies, the influence of temperature on the stability of OVTs has been well researched. Researchers have proposed some methods to advance the temperature stability of OVS, such as temperature control, results compensation, self-healing detection and so on [9,10,11,12]. In [9], Lee improved the temperature stability of an OVT from ±7.0% to ±0.75% within −2~65 °C with dual light path compensation. In [10], the accuracy of an OVT was reported to be improved to ±0.5% within the −40 °C~+60 °C range by introducing a reference voltage for comparison. 

The testing of OVTs based on the above research on temperature characteristics was conducted in the laboratory. The experimental methods usually complied with the relevant standards such as IEC60044-7 [13], which are very different from the actual temperature changes during operation. Many testing results indicate it would be difficult to meet the accuracy requirements of level 0.2 in a wide temperature range. 

The electromagnetic environment and temperature changes during on-site operation are different from the laboratory ones. The accuracy test is usually performed only for a single OVT under laboratory test conditions, whereas three-phase transformers run synchronously in field operation, where the existence of the adjacent phase may cause additional errors [14]. In addition, in the laboratory the temperature experiments are performed according to the testing method recommended in IEC60044-7, in which the temperature gradient is more than that experienced under actual operation and it is unknown whether the testing results in the laboratory can truly reflect the actual performance of field operation, if the on-site operation characteristics are not monitored.

In this paper, we selected OVT prototypes from a company of which multiple OVT products have already been put into operation, and then tested the error characteristics under the three conditions of the laboratory, off-line in the field and during on-line operation. The basic accuracy calibration, temperature characteristic experiments, and the adjacent phase effect experiment were tested in the laboratory. Then the OVT prototypes were transported to the station. The off-line accuracy tests were conducted before operation. Finally, the operation of OVT prototypes was monitored continuously for more than 1 year. The comparison and analysis for the error characteristics of OVT under the three conditions of laboratory, off-line in the field and during on-line operation, are expected to help promote the application of OVTs.

## 2. Principle and Structure of OVT

**Figure 1:**
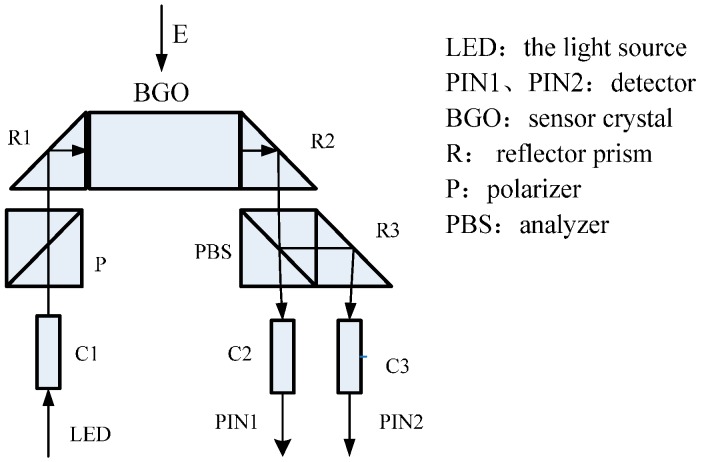
The principle of OVT.

The sensing principle of OVT which has been put into operation is the linear electro-optic effect (Pockels effect), whereby under the action of an external electric field, the refractive index of a sensing crystal changes with the applied electric field, as shown in Figure 1. When linearly polarized light incides on a sensing crystal along some direction, the birefringence phase delay *δ* caused by the Pockels effect is proportional to the applied electric field intensity [15], and:

(1) $$\delta =\frac{\pi l}{\lambda }{n_{0}}^{3}\gamma_{41}E_{k}$$

where *λ* is the wavelength of the input light, *n*_0_ is the refractive index, *γ*_41_ is electro-optic coefficient of the crystal, *l* is the length of the path of the light through the crystal, and *E_k_* is the applied electric field intensity. From Equation (1), the corresponding applied electric field could be obtained by measuring the birefringence phase retardation.

**Figure 2:**
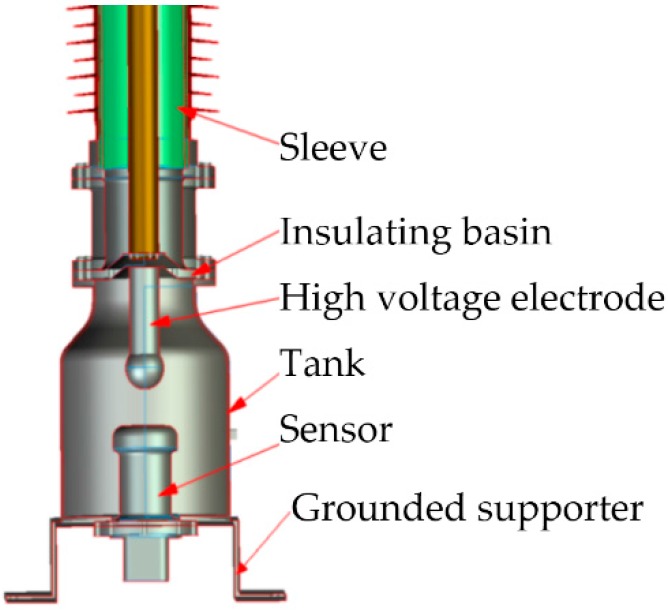
The installation structure diagram of OVT.

The installation structure diagram of an OVT is shown in Figure 2. The voltage sensor was installed inside the bottom tank of the insulator. The voltage to be measured was applied to the voltage sensor through a high voltage electrode in the insulating sleeve. The insulation between the high voltage electrode and ground was reinforced by the insulating basin and SF_6_ gas. The external tank was grounded to shield the external stray electric field. The output light signal of the voltage sensor was led out from the bottom of the cavity, and the signal demodulation was carried out at the bottom grounded supporter or in the control room.

## 3. Testing Scheme 

**Figure 3:**
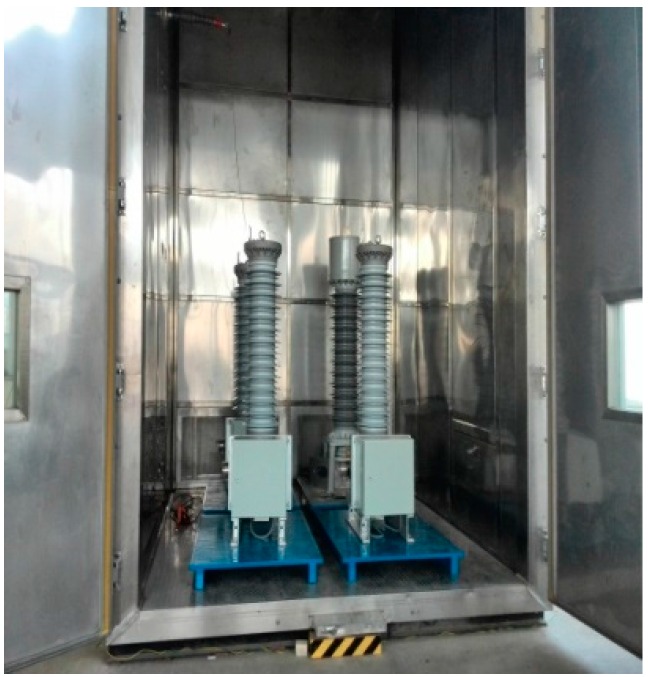
The temperature test in the laboratory.

**Figure 4:**
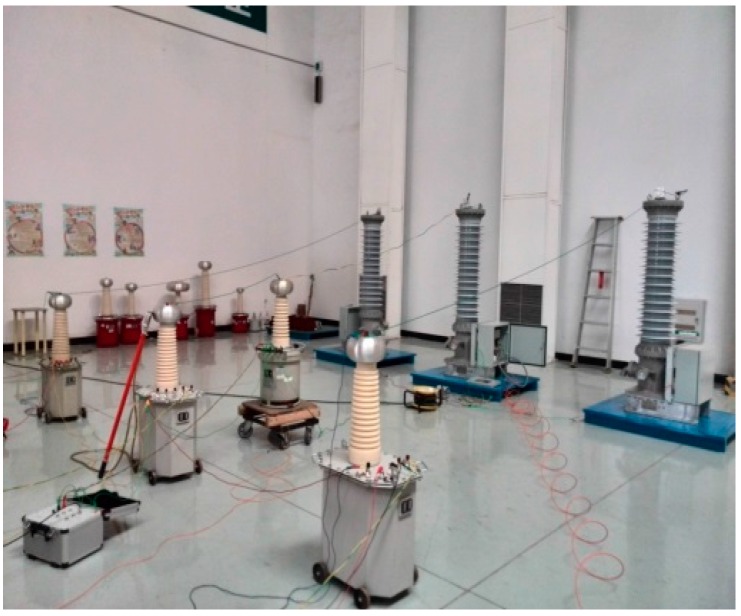
The adjacent phase effect test in the laboratory.

**Figure 5:**
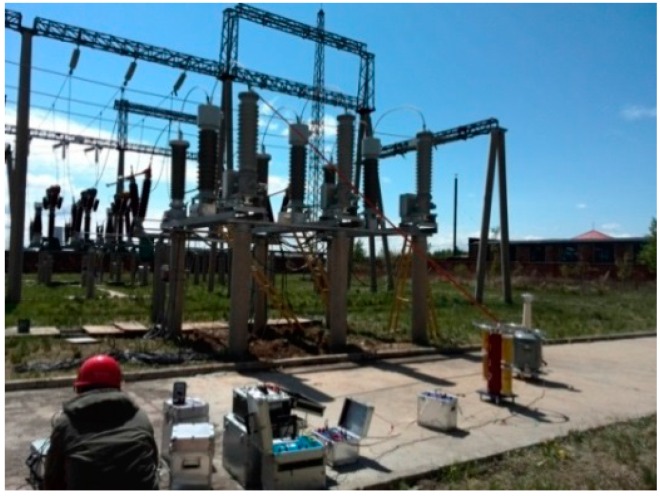
Field off-line accuracy test.

**Figure 6:**
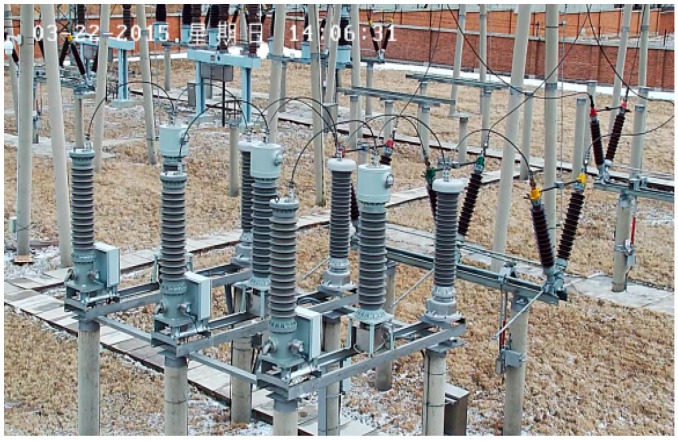
Site installation of the OVT.

**Figure 7:**
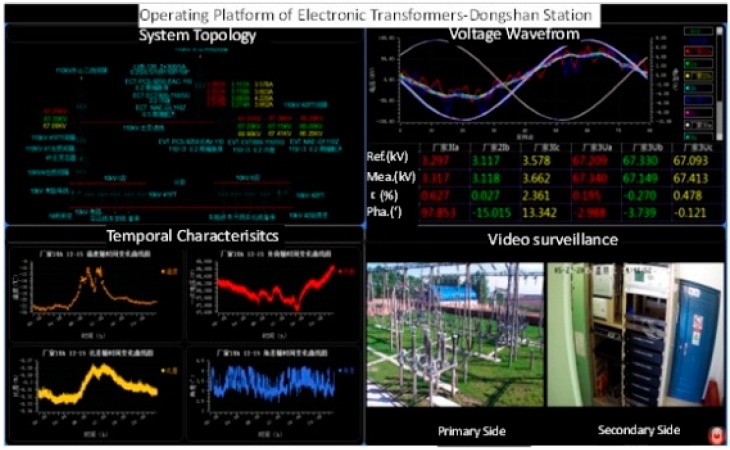
OVT on-line error monitoring system.

Three OVT prototypes based on the structure of Figure 2 were selected with the following parameters: rated voltage of 110/$\sqrt{3}$ kV, rated frequency 50 Hz, accuracy class 0.2, rated secondary output digital quantity 2D41H. The temperature tests (Figure 3) and adjacent phase effect tests (Figure 4) for the OVT prototypes were carried out in laboratory. Then the prototypes were transported to the Dongshan substation in Hegang City (Heilongjiang Province, China) which is an extremely cold area. The field off-line accuracy was tested (Figure 5) before being put into operation. The on-line test results in operation (Figure 6) were transferred to the monitoring system (as shown in Figure 7) located in Wuhan City. Details of the tests are shown in Table 1.

**Figure 8:**
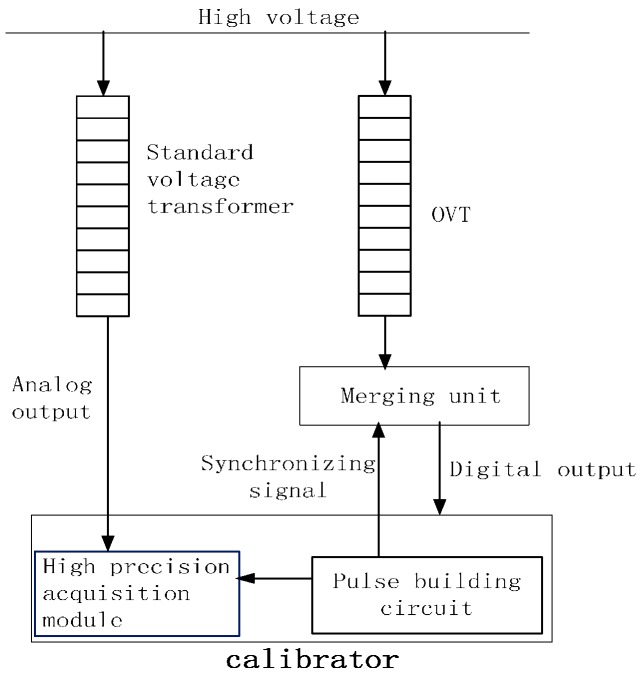
The calibration scheme of OVT in the lab and off-line in the field.

For the laboratory and field off-line accuracy tests, the calibration principle and circuit are shown in Figure 8. The calibration system consists of a standard voltage transformer, high precision acquisition module, pulse forming circuit, industrial control computer, and control and calculation software. The uncertainty of the calibration system was less than 0.05 for ratio error and less than 2 min for phase error. For on-line operation, the accuracy results of OVT were obtained by comparing the output of OVT with the traditional voltage transformer. The accuracy level of traditional voltage transformer was class 0.2. The ratio error *ε* and the phase error *ϕ_e_* can be defined by Equations (2) and (3), respectively:

(2) $$\varepsilon (\%)=\frac{kU_{s}-U_{p}}{U_{p}}\times 100\%$$

(3) $$\varphi_{e}=\varphi_{s}-\varphi_{p}$$

where *U_s_* is the average value of the r.m.s. value in 10 periods from the output of the OVT; *k* is the rated transformation ratio; *U_p_* is the r.m.s. value of primary voltage according to the output of the standard voltage transformer. *ϕ_s_* is the initial phase of *U_s_*, and *ϕ_p_* is the initial phase of *U_p_*.

## 4. Comparison of the OVT Error Characteristics under Three Conditions

In order to ensure the comparability of data, the testing method and the testing equipment in the laboratory were exactly the same as those used in the off-line field tests. For online operation, the output of a traditional voltage transformer was compared as standard data with the same testing method as in the laboratory. After the OVT prototypes were calibrated for the first time, no adjustment or compensation for error were done during the experiments.

### 4.1. Comparison of Error Characteristics of OVT in the Laboratory

**Figure 9:**
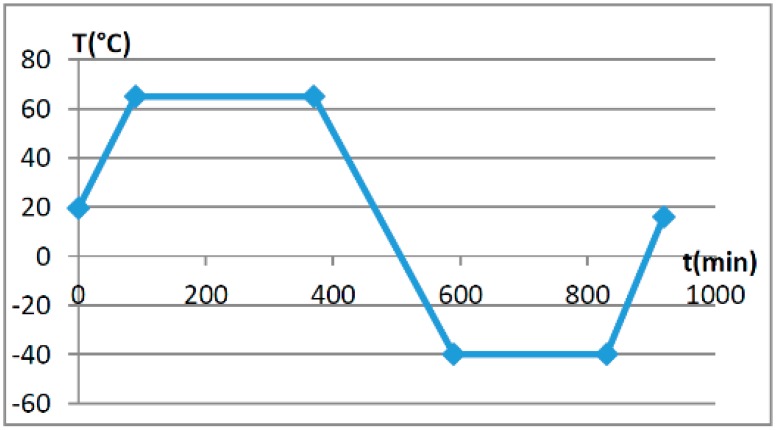
Temperature curve with time.

**Figure 10:**
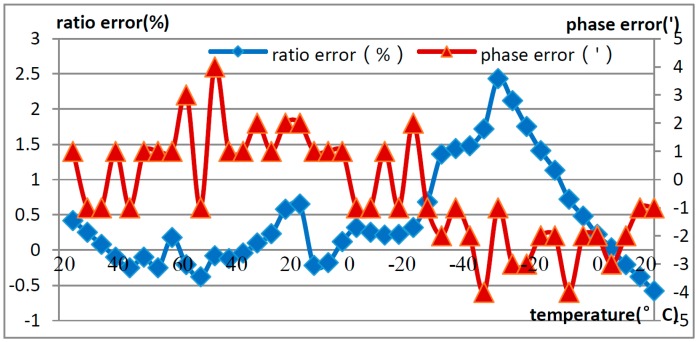
Temperature characteristic of OVTs in the laboratory.

The basic accuracy of the OVT was calibrated and tested under the rated voltage, and then the temperature characteristics were tested. For the temperature characteristic test, three OVT prototypes were placed in a temperature controlled cavity (Figure 3) and subjected to the rated voltage at room temperature (19.5 °C). The temperature changes with time are shown in Figure 9. The prototypes were warmed up to 65 °C at a rate of 10 °C every 20 min and kept at 65 °C for 4 h, then cooled to −40 °C with the same rate and kept at −40 °C for 4 h, and then warmed up to room temperature again with the same rate and kept at room temperature for 4 h. The temperature characteristic of the OVTs was obtained in the temperature range from −40 °C to +65 °C, and phase B OVT testing results are shown in Figure 10, where the ratio error and the phase error were calculated by Equations (2) and (3).

From Figure 10, the ratio error of the OVT is within −0.58% to +2.43% in the temperature range from −40 °C to +65 °C, which is beyond the error limit value of level 0.2, whereas the phase error was within ±10’.

**Figure 11:**
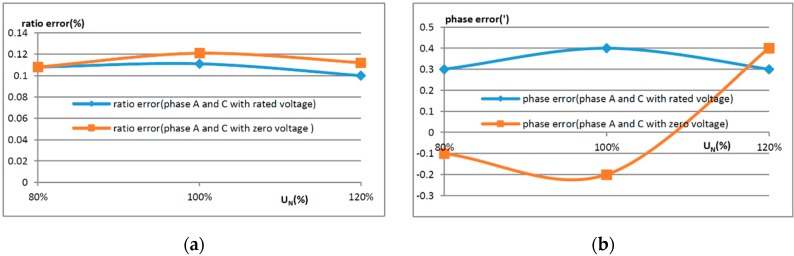
Influence of adjacent phases on OVT. (**a**) Influence of adjacent phases on OVT’s ratio error; (**b**) Influence of adjacent phases on OVT’s phase error.

When the three phase voltage transformers are installed at a certain distance, adjacent phases may interfere with each other. In order to investigate the extent of their mutual influence, three phase OVTs were set at 1 m spacing intervals. The error of the middle phase B was tested when the voltage of phase A and C was applied at zero volts and the rated voltage, respectively. The testing results are shown in Figure 11, where the ratio error and the phase error were calculated by Equations (2) and (3).

From Figure 11, it is shown that the influence of the adjacent phase on OVT is small; the additional ratio error is less than 0.02% and the additional phase error is less than 1’.

### 4.2. Comparison of Error Characteristics of OVT under the Three Conditions

**Figure 12:**
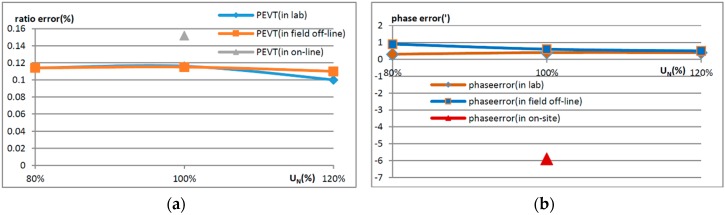
Error comparison of OVT under the three conditions of lab, in the field off-line and during on-line operation. (**a**) The ratio error comparison of OVT; (**b**) The phase error comparison of OVT.

After testing the temperature characteristics and adjacent phase influence in the laboratory, the OVT prototypes were transported to the Hegang substation. The accuracy was tested before being put into operation. The error curves of OVT in the laboratory, in off-line state in the field and in the on-line state are shown in Figure 12. In order to exclude the effect of temperature, the tests in the laboratory and the field off-line tests were carried out at ambient temperature (20 °C). The online operation data was collected during 5 August to 8 August 2014, when the temperature was close to 20 °C. In Figure 12, the ratio error and the phase error were calculated by Equations (2) and (3).

From Figure 12, it can be seen that the errors of OVT in the laboratory and off-line in the field were nearly consistent. The error difference between on-line operation and the laboratory is 0.05%, as the testing results of on-line operation were compared with a traditional voltage transformer. This indicated that the effect of the transportation process and the external electric field caused by field wiring on the OVT could be neglected.

### 4.3. Error Characteristics of OVT in On-Site Operation

**Figure 13:**
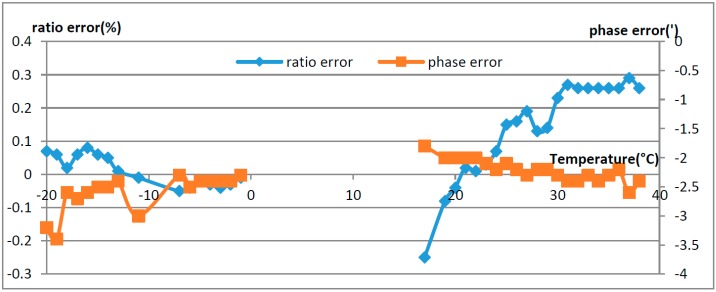
Error comparison of OVT in summer and winter.

The OVT prototypes were put into operation in August 2014, then the data from September 2014 to October 2015 were selected as analysis object. The operation during the two summer and winter seasons which display the greatest temperature difference, were used to illustrate the operational characteristics of the OVTs. The data shown in Figure 13 correspond to August 2015 when the temperatures ranged from 17 °C to 38 °C and December 2014, when the temperature ranged from −20 °C to −1 °C, respectively. In Figure 10, the ratio error and the phase error were calculated using Equations (2) and (3).

From Figure 13, it can be seen that the ratio error of OVT was affected by the temperature. During summer and winter, which display the biggest temperature difference, the ratio error range was from −0.25% to +0.3%, and the phase error was within ± 4′. In summer, the ratio error difference was 0.55%, which is higher than that in winter.

Compared with the temperature characteristics in the laboratory, the temperature characteristics in the field are much better. When the temperature gradient is different, the thermal stress formed in the sensing crystal has a great difference [16,17]. The greater the temperature gradient is, the greater the thermal stress is, and the greater the additional error is. For temperature characteristics in the laboratory the temperature variation rate was 10 degrees every 20 min, but the temperature gradient in the actual runtime environment is far less. Therefore, the on-line operation characteristics of the OVTs were better than the laboratory temperature testing results.

## 5. Discussion

### 5.1. Effect of the Adjacent Phase

The effect of the adjacent phase on a 110 kV OVT with reasonable structure was small. The additional error was within 0.02% and could be ignored for the OVTs of level 0.2.

### 5.2. Differences of the Laboratory and On-Site Field Test Results

The testing results of OVT in the laboratory were in good consistency with those obtained in the field, and the additional error was less than 0.05%. On the one hand, the electric field in the OVT sensors is concentrated, so the accuracy was not sensitive to the wire connection mode; on the other hand, the OVTs were not affected by the vibration of the transportation process.

### 5.3. Differences of Temperature Characteristics

The ratio error range of OVT in the laboratory was −0.58% to +2.43% in the temperature range from −40 °C to 65 °C, and −0.25% to +0.3% during on-site operation in a temperature range from −20 °C to 40 °C. The temperature characteristics of OVT could not meet the 0.2 level accuracy requirements. However, the temperature characteristics during on-site operation were better than those in the laboratory, as the temperature gradient was larger for the laboratory tests than during on-site operation. The temperature characteristics of OVTs were dependent on the temperature gradient.

## Figures and Tables

**Table 1:** Testing cases list for OVTs.

Three Cases	Test Time	Test Site	Remarks
Laboratory	23 March 2014–3 April 2014	Manufacturer	Temperature tests of OVT
	17 April 2014–18 April 2014	State Grid Electric Power Research Institute, Wuhan	Adjacent phase effect tests on OVT
Field off-line	6 May 2014–9 May 2014	Dongshan substation in Hegang City of Heilongjiang Province	The field off-line accuracy tests for OVT
On-line operation	5 August 2014–2016	Dongshan substation in Hegang City of Heilongjiang Province	The online operation of OVT

## Abbreviations

OVT	Optical Voltage Transformer
MDPI	Multidisciplinary Digital Publishing Institute
DOAJ	Directory of Open Access Journals
TLA	Three letter acronym
LD	linear dichroism
